# Psychosocial distress amongst Canadian intensive care unit healthcare workers during the acceleration phase of the COVID-19 pandemic

**DOI:** 10.1371/journal.pone.0254708

**Published:** 2021-08-12

**Authors:** Alexandra Binnie, Kyra Moura, Claire Moura, Frédérick D’Aragon, Jennifer L. Y. Tsang

**Affiliations:** 1 William Osler Health System, Etobicoke, Ontario, Canada; 2 Temerty Faculty of Medicine, University of Toronto, Toronto, Ontario, Canada; 3 School of Medicine, Queen’s University, Kingston, Ontario, Canada; 4 Department of Anesthesiology, Université de Sherbrooke, Sherbrooke, Quebec, Canada; 5 Centre de Recherche du Centre Hospitalier Universitaire de Sherbrooke, Sherbrooke, Quebec, Canada; 6 Niagara Health, St. Catharines, Ontario, Canada; 7 Niagara Regional Campus, Michael G. DeGroote School of Medicine, McMaster University, St. Catharines, Ontario, Canada; Azienda Ospedaliero Universitaria Careggi, ITALY

## Abstract

Intensive care unit healthcare workers (ICU HCW) are at risk of mental health issues during emerging disease outbreaks. A study of ICU HCW from France revealed symptoms of anxiety and depression in 50.4% and 30.4% of workers at the peak of the first wave of the pandemic. The level of COVID-19 exposure of these ICU HCW was very high. In Canada, ICU HCW experienced variable exposure to COVID-19 during the first wave of the pandemic, with some hospitals seeing large numbers of patients while others saw few or none. In this study we examined the relationship between COVID-19 exposure and mental health in Canadian ICU HCW. We conducted a cross-sectional cohort study of Canadian ICU HCW in April 2020, during the acceleration phase of the first wave of the pandemic. Psychosocial distress was assessed using the 12-item General Health Questionnaire (GHQ-12). Participants were asked about sources of stress as well as about exposure to COVID-19 patients and availability of personal protective equipment (PPE). Factors associated with clinically-relevant psychosocial distress were identified. Responses were received from 310 Canadian ICU HCW affiliated with more than 30 institutions. Of these, 64.5% scored ≥ 3 points on the GHQ-12 questionnaire, indicating clinically-relevant psychosocial distress. The frequency of psychosocial distress was highest amongst registered nurses (75.7%) and lowest amongst physicians (49.4%). It was also higher amongst females (64.9%) than males (47.6%). Although PPE availability was good (> 80% of participants reported adequate availability), there was significant anxiety with respect to PPE availability, with respect to the risk of being infected with COVID-19, and with respect to the risk of transmitting COVID-19 to others. In multivariable regression analysis, *Anxiety with respect to being infected with COVID-19* (OR 1.53, CI 1.31–1.81) was the strongest positive predictor of clinically-relevant psychosocial distress while the *Number of shifts with COVID-19 exposure* (OR 0.86, CI 0.75–0.95) was the strongest negative predictor. In summary, clinically-relevant psychosocial distress was identified amongst a majority of ICU HCW during the acceleration phase of the first wave of the COVID-19 pandemic, including those with minimal or no exposure to COVID-19. Strategies to support mental health amongst ICU HCW are required across the entire healthcare system.

## Introduction

Healthcare worker (HCW) distress during emerging disease outbreaks has been attributed to increased workload, shortages of personal protective equipment (PPE), anxiety with respect to being infected, anxiety with respect to transmitting the infection to family and friends, and loss of social supports due to self-isolation and quarantine [1, 2]. During the first wave of the COVID-19 pandemic, studies of HCW in China, Iran, and New York City, reported a high incidence of both anxiety and depression [3–5]. A meta-analysis of 13 studies reported a pooled prevalence of 23.2% for anxiety and 22.8% for depression [6].

Intensive care unit (ICU) HCW may be at higher risk of psychological distress during emerging disease outbreaks. In addition to witnessing the most severe forms of illness, they participate in aerosol-generating medical procedures (AGMP), which can increase the risk of transmitting infection [7]. A study of HCW from 21 ICUs in France during the first wave of the pandemic reported high rates of anxiety (50.4%), depression (30.4%) and peritraumatic dissociation (32%) [8]. Notably, the burden of COVID-19 in these ICUs was high, with each ICU treating an average of 478 COVID-19 patients during the first wave [8]. A study from the United Kingdom reported severe depression in only 6% of ICU staff and severe anxiety in 11%, however the study was conducted in June and July of 2020, several months after the first wave [9]. A study of ICU nurses in the Netherlands, also conducted after the first wave, reported symptoms of anxiety in 27.0% and depression in 18.6% [10].

In Canada, the first wave of the COVID-19 pandemic was characterized by wide geographic variation in case numbers, with some regions experiencing large outbreaks while other regions saw very few cases. In this study we surveyed Canadian ICU HCW during the acceleration phase of the first wave of the pandemic to identify risk factors for clinically-relevant psychosocial distress in the context of accelerating infections and variable COVID-19 exposure levels.

## Materials and methods

### Survey design

The survey was designed as the entry questionnaire for a longitudinal study of exposure and transmission risks for COVID-19 infection amongst Canadian ICU HCW. It was built in an expedited fashion to capture data during the acceleration phase of the first wave of the pandemic. Based on literature review, four domains were identified as potential contributors to HCW psychosocial distress in emerging infectious disease outbreaks: (1) demographics (2) exposure to infected patients, (3) access to PPE and (4) anxieties regarding availability of PPE, risk of infection, and risk of transmitting infection to others. To assess psychosocial distress, participants completed the General Health Questionnaire 12-item scale (GHQ-12), a screening tool that focuses on breaks in normal functioning rather than life-long traits [11]. Participants were also asked to rate the level of stress associated with six “life domains”: *work life*, *home life*, *finances*, *physical health*, *mental health*, and *family health*, on a scale of 0–100. The complete survey is shown in **S1 Appendix** and the survey design methodology is described in **S2 Appendix**.

### Survey administration

The survey was administered online through the secure Qualtrics platform (www.qualtrics.com). The target population was Canadian ICU HCW including physicians, registered nurses, respiratory therapists, and allied healthcare. The survey link was disseminated through online advertisement via the Canadian Critical Care Trials Group, the Canadian Community ICU Research Network, the Canadian Critical Care Society, and the Critical Care Nurses Association and related social media groups. Distribution began on April 6^th^, 2020 and all responses were received by April 30^th^, 2020, prior to the peak of the first wave of the pandemic in Canada [12].

### Data analysis

Data were described as number and percentage for categorical variables and mean and standard deviation or median and interquartile range for continuous variables. Between-group differences were assessed using the student t-test or Wilcoxon rank-sum test (continuous variables) and Chi-square or Kruskal-Wallis tests (categorical variables). Correlations were assessed using Pearson’s (continuous) or Spearman’s (categorical) correlations.

Response categories on the GHQ-12 questionnaire were coded using the 0-0-1-1 scoring method [11]. A threshold of ≥ 3 points on the GHQ-12 was identified as an appropriate cutoff for mental health diagnosis screening [13, 14] and a marker of clinically-relevant psychosocial distress [15, 16]. Predictors of clinically-relevant psychosocial distress (GHQ-12 ≥ 3) were identified using univariate logistic regression models. Variables were chosen for inclusion in multivariate logistic regression based on a p-value < 0.2 in univariate regression. Forward and backward stepwise regression was performed to achieve the lowest Akaike Information Criteria (AIC) with each variable in the final model showing a p-value < 0.05. All analysis was performed using R software v 3.6.2 [17].

### Ethical consideration

Ethics approval was obtained from the Hamilton Integrated Research Ethics Board (HiREB #10790) and the Centre Hospitalier Universitaire de Sherbrooke (#MP-31-2021-3704).

## Results

A total of 310 ICU HCW completed the survey in April, 2020. Although respondents were not required to provide an institutional affiliation, 130 (42%) provided email addresses from Canadian hospitals, medical systems, or universities, representing 33 different institutions. The majority of participants were working in the province of Ontario (78%), and the remainder in Quebec (7.7%), Alberta (7.1%), Manitoba (6.1%), the Atlantic provinces (0.6%) and British Columbia (0.3%). Registered nurse (RN) was the most common profession (47.7%), followed by physician (26.7%), respiratory therapist (RT) (14.2%), and allied health (11.3%) (Table 1). The mean age of participants was 40 years (SD = 9.7) A majority of respondents were female (73.5%).

**Table 1:** Participant characteristics.

	Total	Registered Nurse	Physician	Respiratory therapist	Allied Health
	N = 310	N = 148	N = 83	N = 44	N = 35
Sex					
Female	228 (73.5%)	135 (91.2%)	35 (42.2%)	28 (63.6%)	30 (85.7%)
Male	82 (26.5%)	13 (8.8%)	48 (57.8%)	16 (36.4%)	5 (14.3%)
Age (years)					
Mean (SD)	40 (± 9.7)	37 (± 10)	43 (± 8.9)	39 (± 6.9)	44 (± 9.0)
Years of experience					
Mean (SD)	14 (± 9.5)	12 (± 9.9)	16 (± 9.6)	13 (± 6.7)	17 (± 8.9)
Province					
Ontario	242 (78.1%)	126 (85.1%)	47 (56.6%)	41 (93.2%)	28 (80.0%)
Quebec	24 (7.7%)	7 (4.7%)	12 (14.5%)	1 (2.3%)	4 (11.4%)
Alberta	22 (7.1%)	10 (6.8%)	8 (9.6%)	1 (2.3%)	3 (8.6%)
Manitoba	19 (6.1%)	5 (3.4%)	13 (15.7%)	1 (2.3%)	0 (0%)
British Columbia	1 (0.3%)	0 (0%)	1 (1.2%)	0 (0%)	0 (0%)
Nova Scotia	1 (0.3%)	0 (0%)	1 (1.2%)	0 (0%)	0 (0%)
Newfoundland and Labrador	1 (0.3%)	0 (0%)	1 (1.2%)	0 (0%)	0 (0%)

### Psychosocial stress

The mean GHQ-12 score for all participants was 3.8 (median = 4, SD = 2.8). The questions that gave the most frequently abnormal responses were: “Have you felt constantly under strain?” (positive response, 78.7%), “Have you lost much sleep over worry?” (positive response, 54.2%), and “Have you been able to enjoy your normal day-to-day activities?” (negative response, 51.9%). Questions that gave the fewest abnormal responses were “Have you been thinking of yourself as a worthless person” (positive response, 7.4%), “Have you felt capable of making decisions about things?” (negative response, 8.1%), and “Have you felt that you could not overcome your difficulties?” (positive response, 10.3%).

**Fig 1:**
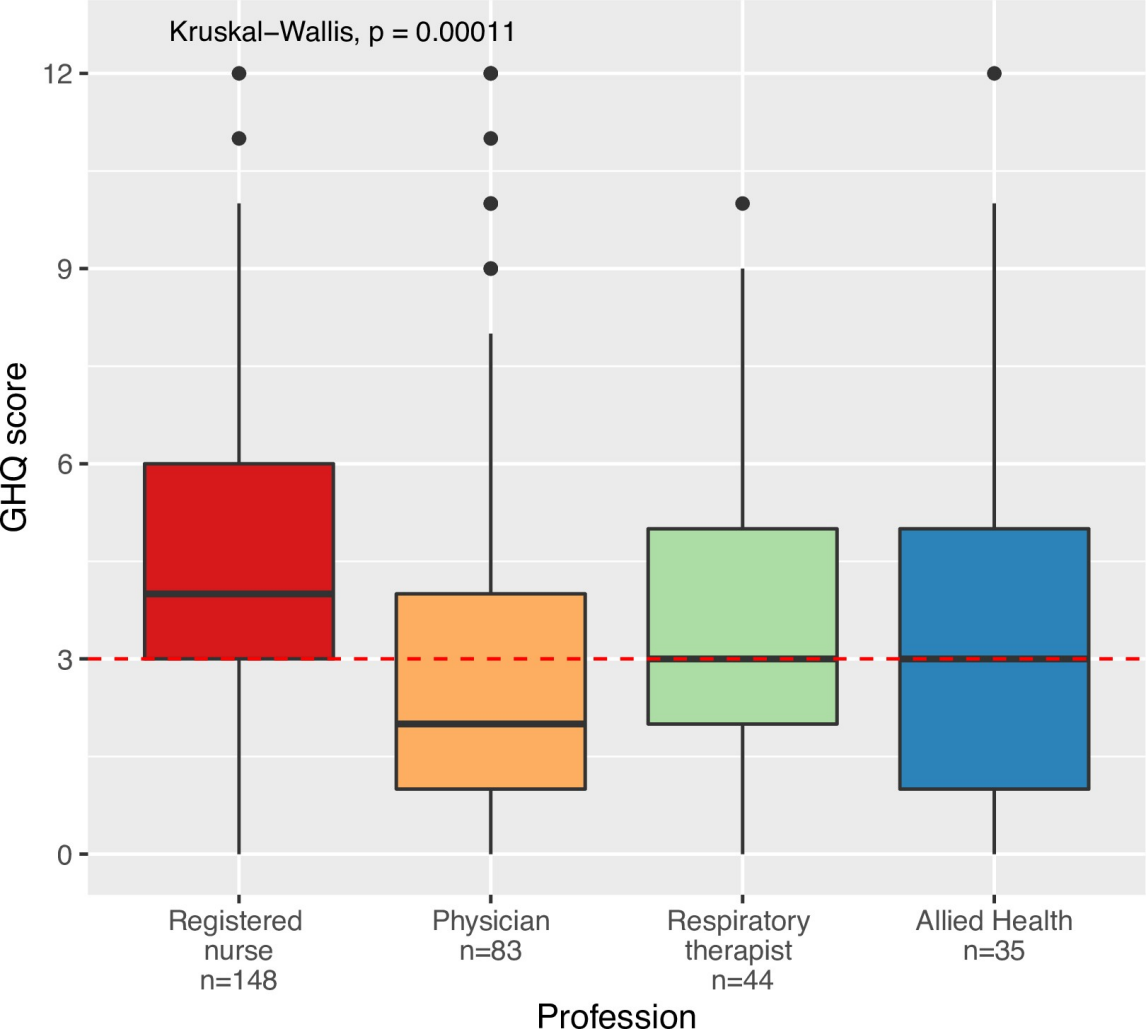
GHQ-12 score relative to profession. The dotted line represents a GHQ-12 score of 3, which is the cutoff for clinically-relevant psychosocial distress.

GHQ-12 scores varied significantly by profession (p<0.001) (Fig 1). RNs had the highest GHQ-12 scores at a mean of 4.4 (median = 4, SD = 2.6) while physicians had the lowest at a mean of 3.0 (median = 3, SD = 3.0). RTs and allied health were intermediate at 3.6 (median = 4, SD = 2.4) and 3.6 (median = 4, SD = 2.9), respectively. On pairwise t-testing, RNs scored significantly higher than physicians (p<0.0001).

GHQ-12 scores also varied by sex, with females averaging 4.2 (median = 4, SD = 2.8) and males 2.9 (median = 3, SD = 2.6) (p<0.0001). When subdivided by profession, the difference between females and males was significant for RNs but not for other professions (S1 Fig).

Although age is a risk factor for severe COVID-19 infection [18], there was no significant correlation between age and GHQ-12 scores (r = -0.07, p = 0.20). Years of experience, which was strongly correlated with age (r = 0.88, p<0.001), also showed no correlation with GHQ-12 scores (r = -0.07, p = 0.22).

### Incidence of clinically relevant psychosocial distress

Amongst the 310 participants, 200 (64.5%) reported a GHQ-12 score ≥ 3 points, indicating clinically-relevant psychosocial distress. Profession was significantly associated with clinically-relevant psychosocial distress (p<0.01). On pairwise testing, RNs (75.7%) were more likely to report clinically relevant psychosocial distress than were physicians (49.4%) (p<0.05). RTs and allied health were intermediate at 61.4% and 57.1%, respectively. The prevalence of clinically-relevant psychosocial distress was higher amongst female respondents (64.9%) than males (47.6%, p = 0.009).

### Sources of stress

**Fig 2:**
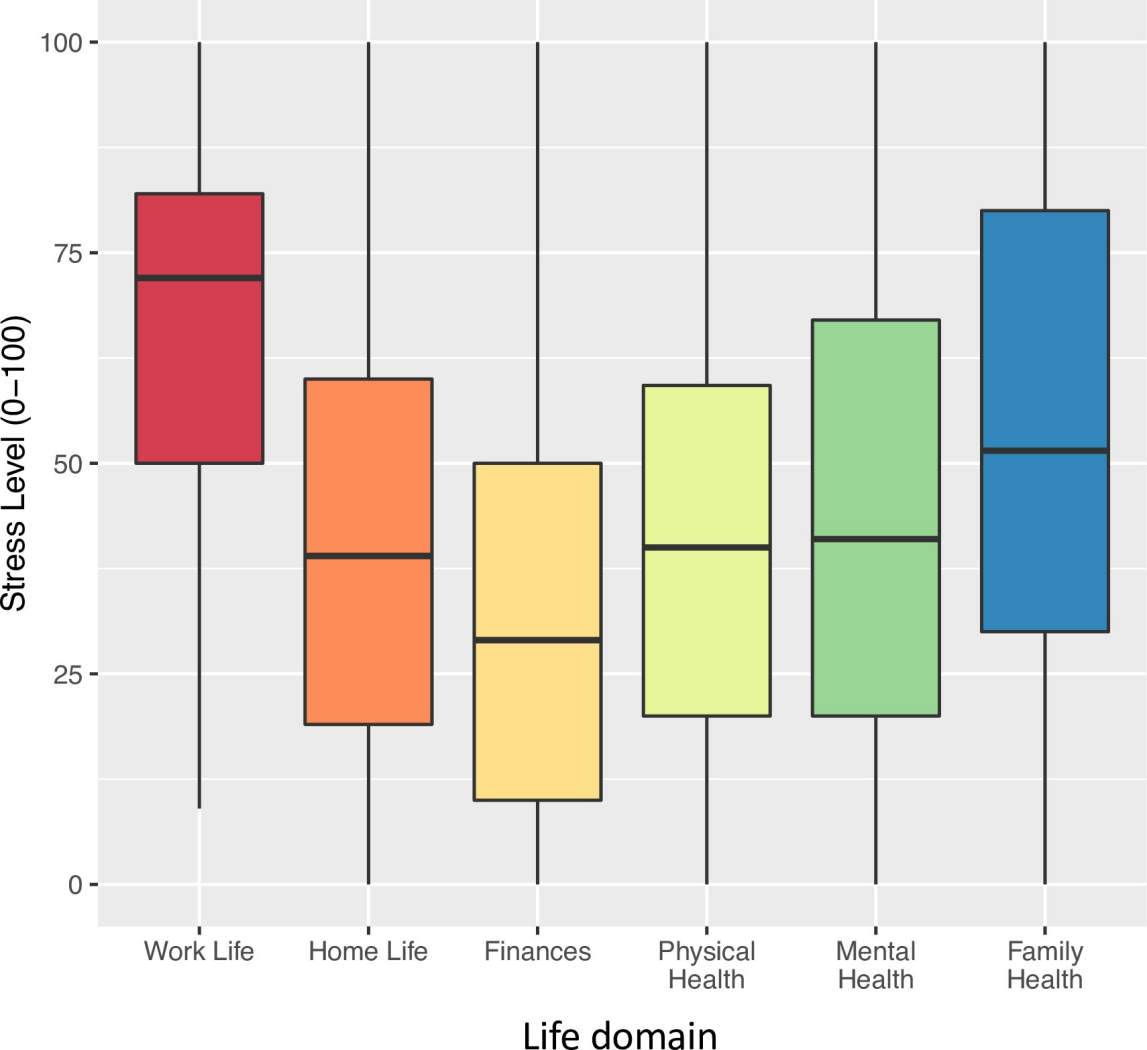
Stress levels attributed to specific life domains. Participants were asked to rate their level of stress relative to each “life domain” on a scale from 0–100.

To identify sources of stress, participants were asked to rate the level of stress related to six “life domains” on a scale from 0–100: *work life*, *home life*, *finances*, *physical health*, *mental health*, and *family health* (Fig 2). *Work life* was rated as the most stressful domain by all professions, with a mean score of 67/100 (SD = 24). RNs (70 ± 24) and RTs (70 ± 22) rated *work life* as significantly more stressful than did physicians (61 ± 26) (p<0.05). The second most stressful domain was *family health* (mean = 54, SD = 28). *Finances* was the least stressful domain with a score of 32 (SD = 24).

On correlation analysis, stress levels in all domains, with the exception of *finances*, were positively correlated with GHQ-12 scores. The strongest correlations were for *mental health* (r = 0.58, p<0.0001) and *work life* (r = 0.51, p<0.0001).

### Measures of COVID-19 exposure

The number of COVID-19 patients reported by participants in their ICUs varied significantly from zero COVID-19 patients (9.6%) to more than 20 patients (1.8%). Almost half, however, reported 1–5 COVID-19 patients (43.9%) in their ICU with the remainder reporting 6–10 patients (22.9%) or 11–20 patients (21.8%). Only 19.5% of respondents reported that critically-ill COVID-19 patients were being cared for outside the regular ICU at their hospital, suggesting that ICU capacity had been exceeded.

With respect to individual exposure, participants reported an average of 3.6 (SD = 1.9) shifts worked in the previous week, of which they had exposure to COVID-19 patients on 2.1 shifts (SD = 2.1 shifts). More than a quarter of participants (29%) reported no exposure to COVID-19 patients in the previous week while 27.4% reported ≥ 4 shifts with exposure.

**Fig 3:**
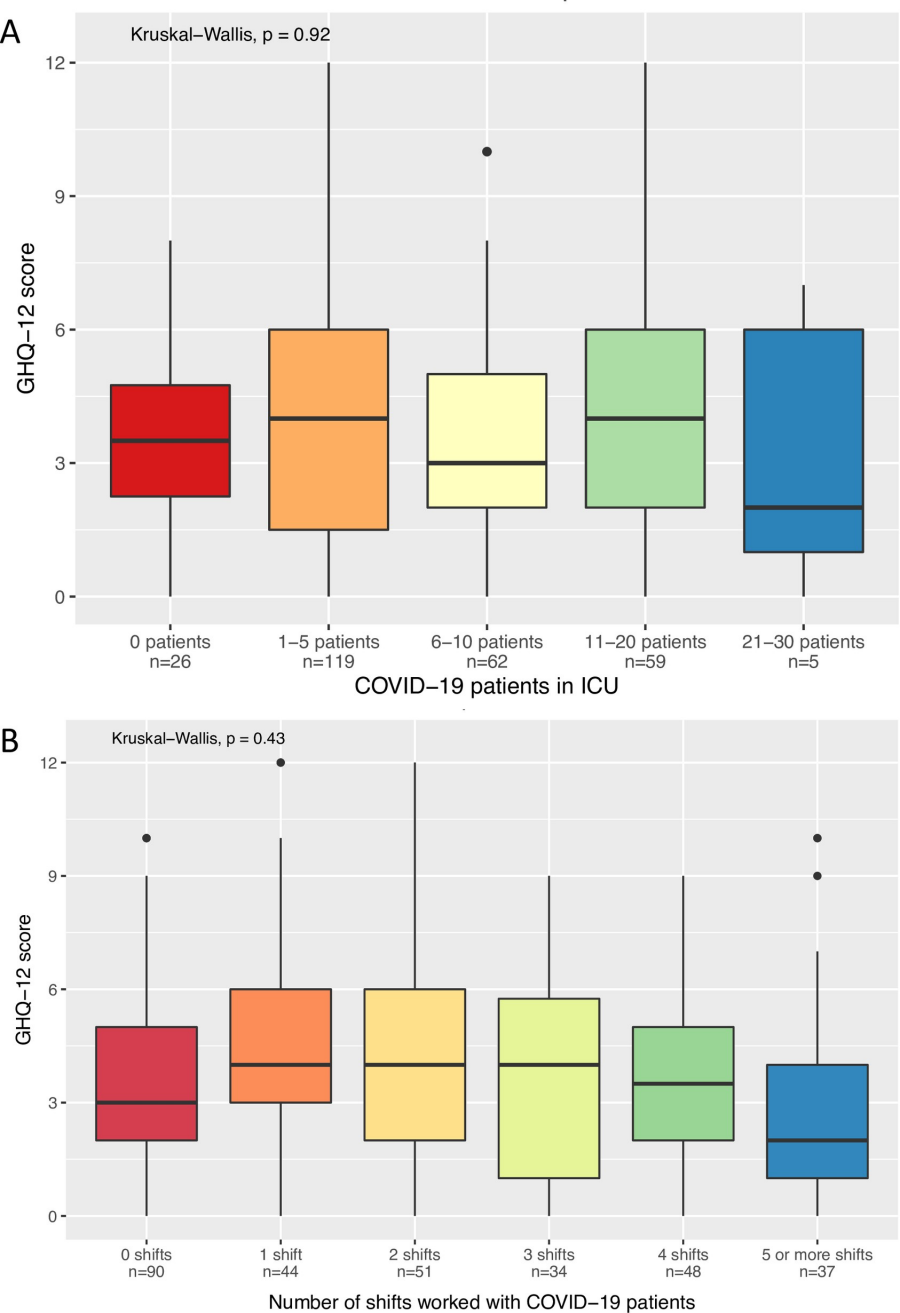
GHQ-12 score relative to markers of COVID-19 exposure. (A) GHQ-12 score relative to number of COVID-19 patients in the participant’s ICU in the previous week. (B) GHQ-12 scores relative to number of shifts worked with COVID-19 patients in the previous week.

**Fig 4:**
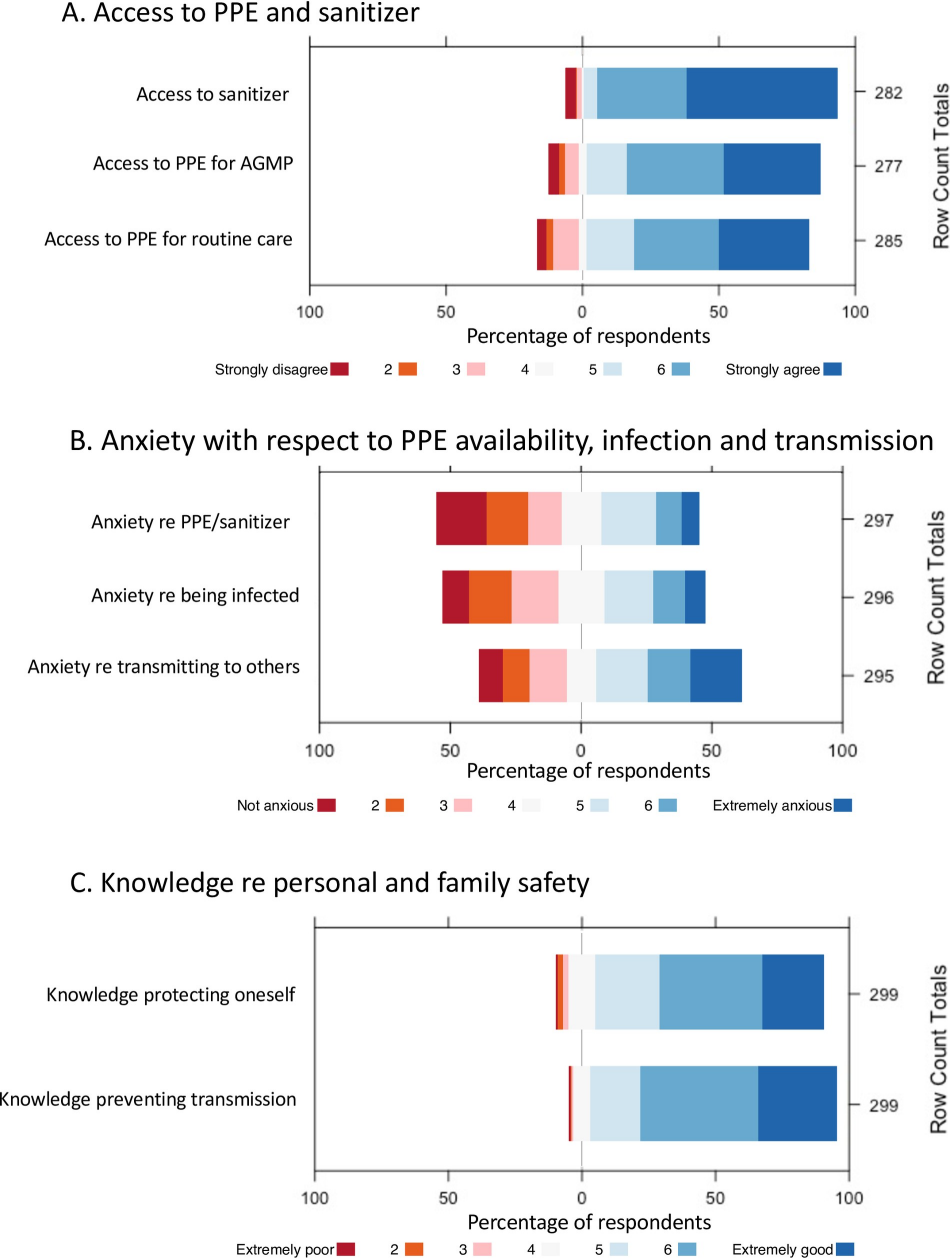
Responses to 7-point Likert questions. Diverging stacked bar charts of 7-point Likert responses re (A) Access to sanitizer, PPE for routine patient care and PPE for AGMP, (B) Anxiety with respect to PPE availability, risk of being infected with COVID-19, and risk of transmitting COVID-19 to others and (C) Knowledge re protecting oneself from COVID-19 and preventing transmission of COVID-19 to others.

In correlation analysis, markers of individual COVID-19 exposure did not correlate with GHQ-12 scores. In particular, there were no significant correlations between GHQ-12 scores and (1) the number of COVID-19 patients in the participant’s ICU (r = -0.01, p = 0.86) (Fig 3), (2) the number of shifts worked in the previous week (r = -0.02, p = 0.73), (3) the number of shifts worked with COVID-19 patients in the previous week (r = -0.05, p = 0.41) (Fig 3), and (4) whether critically-ill COVID-19 patients were being cared for outside the regular ICU in the participant’s hospital (r = 0.02, p = 0.69).

Respondents were also asked about exposure to procedures that are aerosol-generating (AGMP) or that might increase the risk of COVID-19 transmission due to close contact with the patient. These included intubation, extubation, bag-mask-ventilation, tracheostomy, bronchoscopy, proning/supinating, and cardiac arrest resuscitation. The median number of exposures to these procedures in COVID-19 patients in the previous week was 1 (IQR: 0–3), however almost half of respondents (49%) reported no exposures while 14.4% reported 5 or more exposures. Proning/supinating was the most common procedure for all professions and intubation was the second most common procedure for RNs, RTs and physicians (S2 Fig). In correlation analysis, no significant correlation was observed between GHQ-12 scores and the number of procedures (r = -0.08, p = 0.21) (S2 Fig).

### PPE availability

**Fig 5:**
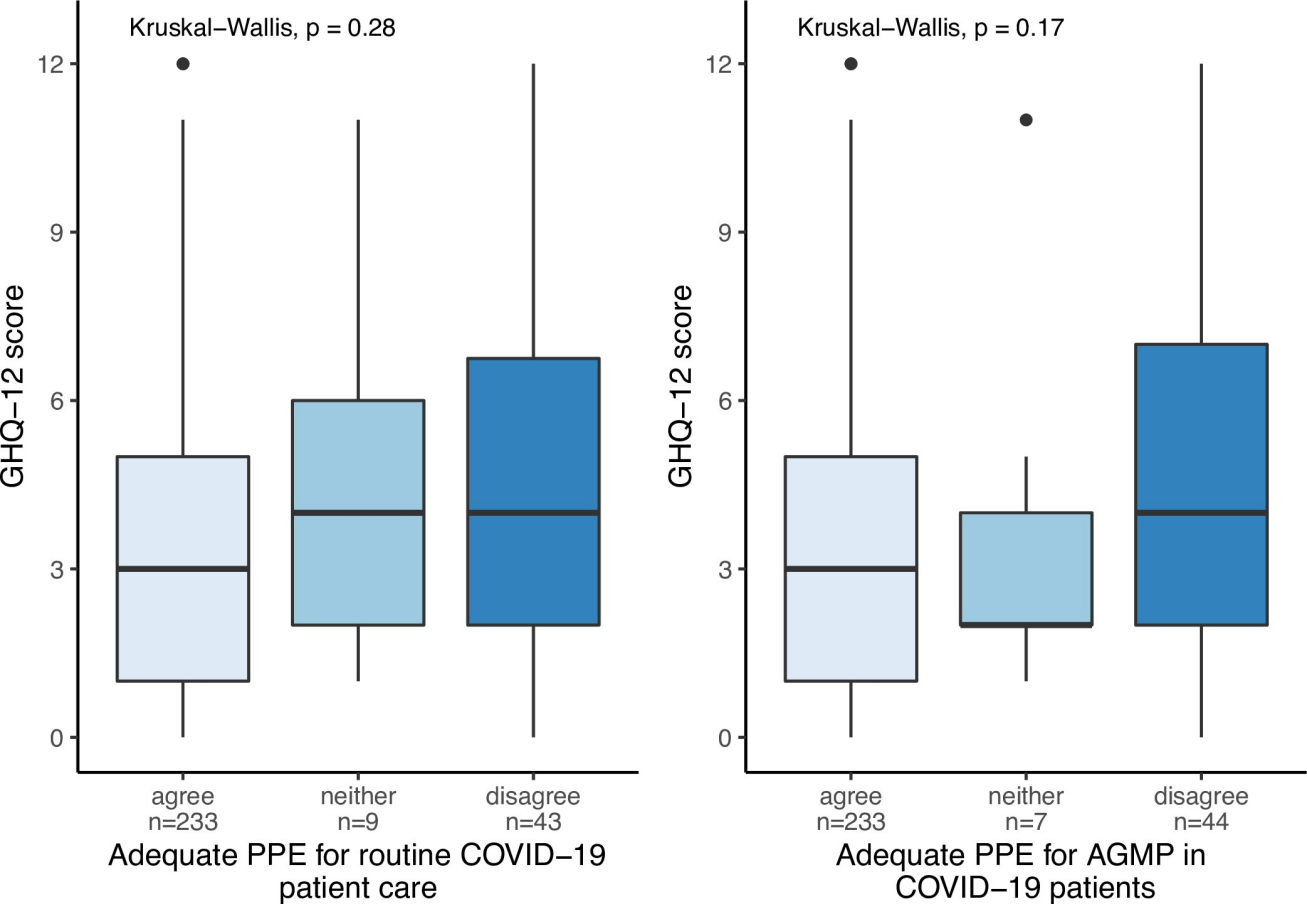
GHQ-12 scores relative to PPE availability. GHQ-12 scores relative to (A) access to PPE for routine patient care and (B) access to PPE for AGMP. Participants were subdivided into “disagree” (1–3 points on a 7-point Likert scale), “neutral” (4 points) and “agree” (5–7 points).

Respondents were asked about access to PPE in their ICUs (Fig 4A). PPE availability was generally good, with 81.8% agreeing with the statement “in the past week, I have always had access to appropriate PPE for *routine patient care*”, and 85.9% agreeing that “in the past week, I have always had access to appropriate PPE for *aerosol-generating medical procedures* in COVID-19 patients” (S1 Fig). Participants who reported inadequate access to PPE also reported higher GHQ-12 scores, however the difference was not statistically significant (Fig 5).

### Anxiety with respect to PPE availability, risk of infection, and risk of transmission

**Fig 6:**
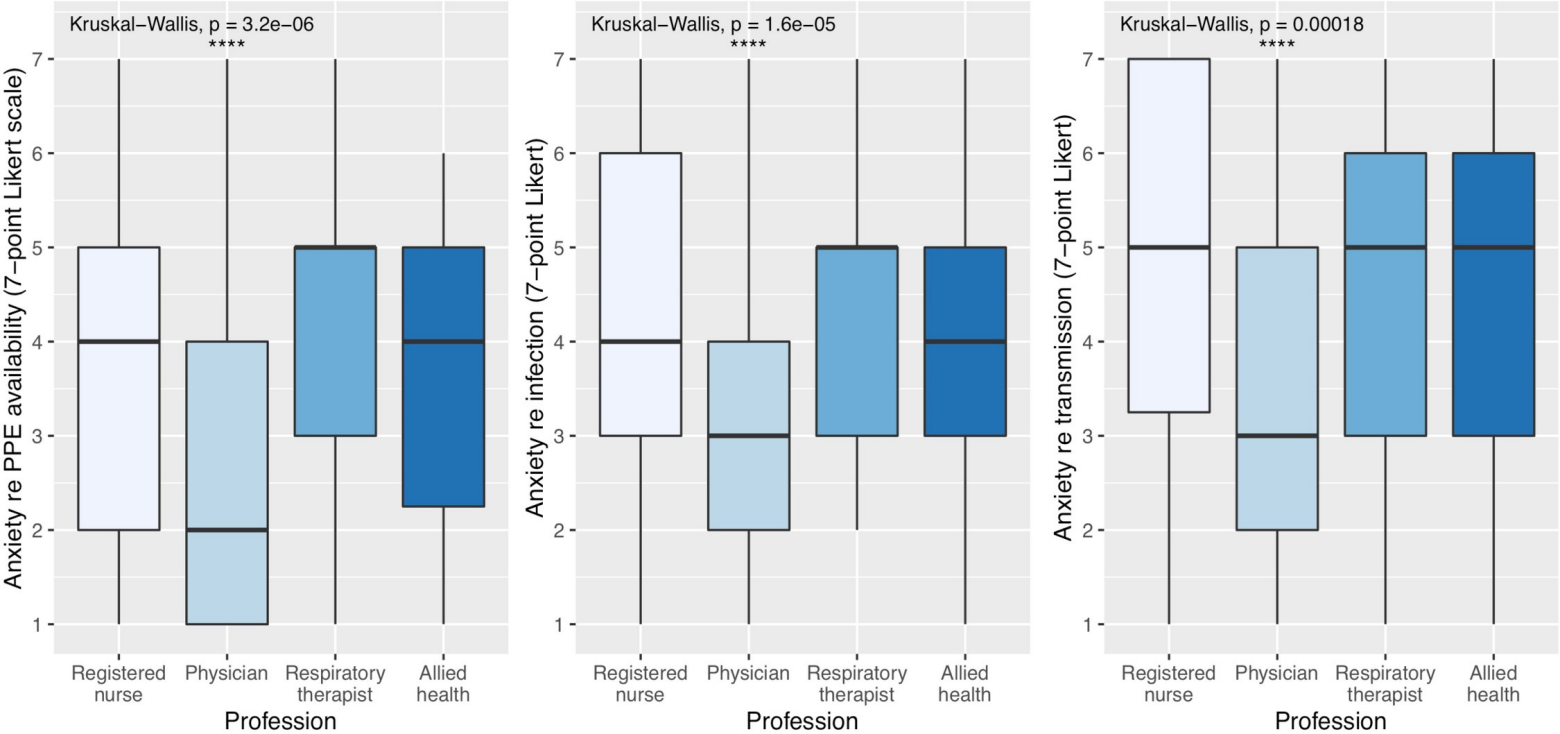
Boxplot of anxiety levels subdivided by profession. (A) Anxiety with respect to PPE availability. (B) Anxiety with respect to risk of becoming infected with COVID-19. (C) Anxiety with respect to risk of transmitting COVID-19 to family or loved ones.

Respondents were asked to rate their *Anxiety with respect to PPE availability* on a 7-point Likert scale from “Not anxious (1)” to “Extremely anxious (7)” (Fig 4B). Amongst the different professions, RTs reported the highest *Anxiety with respect to PPE availability* at 4.5 (SD = 1.6), relative to 3.8 for RNs (SD = 1.9), 3.7 for allied health (SD = 1.6) and 2.7 for physicians (SD = 1.7) (Fig 6). Pairwise testing confirmed that physicians reported less *Anxiety with respect to PPE availability* than did other professionals (p <0.05 for allied health, p <0.001 for RNs, and p <0.0001 for RTs) (Fig 6). *Anxiety with respect to PPE availability* correlated negatively with Years of Experience (r = -0.17, p<0.01) and positively with GHQ-12 scores (r = 0.25, p < 0.0001).

Participants were also asked to rate their *Anxiety with respect to being infected with COVID-19* (Fig 4B). RTs reported the highest *Anxiety with respect to being infected with COVID-19* at 4.4 (SD = 1.5), relative to 4.1 (SD = 1.8) for RNs, 4.0 (SD = 1.6) for allied health and 3.0 (SD = 1.6) for physicians. Pairwise testing revealed that physicians reported less *Anxiety with respect to being infected with COVID-19* than did other professionals (p <0.05 for allied health, p <0.001 for RNs, and p <0.001 for RTs) (Fig 6). *Anxiety with respect to being infected with COVID-19* correlated negatively with Years of Experience (r = -0.18, p<0.01) and positively with GHQ-12 scores (r = 0.34, p <0.0001).

Finally, participants were asked to rate their *Anxiety with respect to transmitting COVID-19 to others* (Fig 4B). RNs reported the highest *Anxiety with respect to transmitting COVID-19 to others* at 4.9 (SD = 1.9), relative to 4.6 (SD = 1.8) for allied health, 4.5 (SD = 1.8) for RTs and 3.7 (SD = 1.9) for physicians. Pairwise testing revealed that physicians reported less anxiety than did RNs (p <0.0001) (Fig 6). *Anxiety with respect to transmitting COVID-19 to others* also correlated negatively with Years of Experience (r = -0.18, p<0.01) and positively with GHQ-12 scores (r = 0.28, p <0.000001).

### Knowledge with respect to prevention of infection and transmission

Participants were asked to rate their *Knowledge with respect to protecting themselves from COVID-19* and their *Knowledge with respect to preventing transmission of COVID-19 to family and loved ones* on a 7-point Likert scale from “Extremely poor (1)” to “Extremely good (7)” (Fig 4C). Participants rated their *Knowledge with respect to protecting themselves* at 5.9 (SD = 1.0) and their *Knowledge with respect to preventing transmission* at 5.6 (SD = 1.2). *Knowledge with respect to protecting oneself* varied by profession (p<0.001), with physicians reporting higher knowledge scores than did allied health (p<0.01). *Knowledge with respect to preventing transmission to family and loved ones* did not vary by profession.

### Multivariable regression analysis

Univariate logistic regression indicated that the following variables were predictive of clinically-relevant psychosocial distress: *Sex*, *Profession*, *Anxiety with respect to PPE availability*, *Anxiety with respect to being infected with COVID-19*, and *Anxiety with respect to transmitting COVID-19 to others* (S1 Table). In multivariable logistic regression analysis, only two variables were significantly associated with GHQ-12 scores. These were *Anxiety with respect to being infected with COVID-19* (OR 1.53, CI 1.31–1.81), which was a positive predictor, and *Number of shifts with COVID-19 exposure* (OR 0.86, CI 0.75–0.95), which was a negative predictor (Table 2).

**Table 2:** Results of multivariate logistic regression analysis of factors predicting clinically-relevant psychosocial distress (GHQ-12 ≥ 3).

Characteristic	OR	95% CI	p-value	p-adj
Weekly COVID-19 shifts	0.86	0.75, 0.98	0.024	0.024
Anxiety re personal risk of infection	1.53	1.31, 1.81	<0.001	<0.001

OR = odds ratio, CI = confidence interval, p-adj = p-value adjusted for multiple testing.

## Discussion

At the peak of the first wave of the COVID-19 pandemic, 64.5% of Canadian ICU HCW in this cross-sectional cohort study displayed symptoms of clinically-relevant psychosocial distress. RNs were at higher risk of psychosocial distress than physicians, while RTs and allied health were intermediate. Female sex was associated with increased risk of clinically-relevant psychosocial distress, particularly amongst RNs. Age and years of experience were not associated with clinically-relevant psychosocial distress. Participants reported that *work life* was their most significant source of stress followed by *family health*.

In multivariable regression analysis, the best predictors of clinically-relevant psychosocial distress were *Anxiety with respect to being infected with COVID-*19 and *Number of COVID-19 shifts worked in the previous week*. Other markers of COVID-19 exposure such as the number of COVID-19 patients in the ICU and the number of procedures performed in COVID-19 patients were not predictive. Interestingly, the *Number of shifts worked with COVID-19 patients in the previous week* was a negative predictor of psychosocial distress, indicating that higher exposure to COVID-19 patients was associated with lower levels of psychosocial distress. Moreover, participants who reported no COVID-19 patients in their ICUs showed similar levels of psychosocial distress to other participants [15].

During the 2003 Severe Acute Respiratory Syndrome (SARS) outbreak in Toronto, a study of local HCW reported that 29% met criteria for clinically-relevant psychosocial distress [15]. Amongst registered nurses this was 45%. Levels of clinically-relevant psychosocial distress in the current study were much higher, with 67% of participants testing positive. This difference may reflect the unique challenges posed by the COVID-19 outbreak, including shortages of PPE, nationwide community spread, and ubiquitous media and social-media coverage. It may also reflect differences between the ICU and other hospital departments, where exposure to the virus is less likely to occur. Finally, it may reflect the timing of the current study, which took place during the acceleration phase of the first wave of the pandemic, when there was considerable uncertainty around the risk of infection to HCW.

While the GHQ-12 instrument does not diagnose specific mental health conditions, a score of ≥3 points has good specificity (74–88%) and sensitivity (74–80%) for psychiatric disease, particularly anxiety and mood disorders [14, 19]. Thus, the results of this study are consistent with other studies showing a high prevalence of mental health issues amongst ICU HCW during the first wave of the COVID-19 pandemic [4]. A study of French ICU HCW, for example, reported anxiety and depression in 50.4% and 30.4% of respondents at the peak of the first wave [8]. Similarly, a study from China reported symptoms of anxiety and depression in 65.9% and 58.7% of ICU HCW during the deceleration phase of the first wave [20]. Finally, a study of ICU HCW in New York City revealed symptoms of anxiety and depression in 48% and 33% of respondents just after the peak of the first wave [21]. In spite of variable exposure to COVID-19, Canadian ICU HCW showed similar levels of mental health issues to ICU HCW in these early hotspots. [14, 19] Our results highlight the broad psychological impact of the pandemic on ICU HCW during the first wave of the pandemic, even those with little exposure to COVID-19 patients.

Follow-up studies of HCW in Toronto after SARS revealed that workers in hospitals that treated SARS patients were more likely to experience burnout, psychosocial distress, and post-traumatic stress symptoms [22, 23]. They were also more likely to reduce their work hours, miss work due to stress or illness, and increase their use of tobacco, alcohol or other problematic behaviours [22]. Measures to minimize the long-term effects of psychosocial distress on ICU HCW are needed in order to protect the mental health of ICU HCW and prevent burnout [24–28]. These measures may be equally important in regions with little exposure to COVID-19 as they are in areas with high exposure.

Our study has several limitations. Participants were self-selected and may not represent Canadian ICU HCW as a whole. However, the study was advertised as a study of infection and transmission risks rather than mental health, which may have mitigated this self-selection effect. Participants were weighted towards the province of Ontario, with relative underrepresentation of other Canadian provinces. Finally, some professions (e.g. dietician, physiotherapist, pharmacist) were grouped together under “allied health”, resulting in a loss of data.

## Conclusions

In summary, nearly two thirds of Canadian ICU HCW experienced clinically-relevant psychosocial distress during the acceleration phase of the first wave of the pandemic, irrespective of COVID-19 exposure. Registered nurses were more affected than other professionals. *Anxiety with respect to risk of being infected with COVID-19* was an important modifiable risk factor and should be a focus for workplace interventions during this and future pandemics. Our results demonstrate the need for proper appreciation of HCW services, early recognition of psychological symptoms, and provision of post-pandemic care for ICU HCW mental health.

## Supporting information

S1 Fig GHQ-12 scores relative to profession and sex. Boxplots of GHQ-12 scores relative to profession and subdivided by sex. P-values compare females and males for each professional group.
(TIF)

S2 Fig Procedures in COVID-19 patients. (A) Number of procedures per shift in COVID-19 patients performed by respondents in the previous week. (B) Plot of number of procedures performed in COVID-19 patients in the previous week relative to GHQ-12 score.
(TIF)

S1 Table Results of univariate logistic regression for clinically-relevant psychosocial distress (GHQ-12 ≥ 3). (PDF)

S1 Appendix Study survey. The complete study survey is shown with the exception of the GHQ-12 questionnaire.
(PDF)

S2 Appendix Survey design methodology. (PDF)

S1 Dataset (CSV)
