# Supplementary figures and images for: Psychosocial distress amongst Canadian intensive care unit healthcare workers during the acceleration phase of the COVID-19 pandemic
Source: PLoS One. 2021 Aug 12;16(8):e0254708. doi: 10.1371/journal.pone.0254708 (PMC8360506; doi:10.1371/journal.pone.0254708)

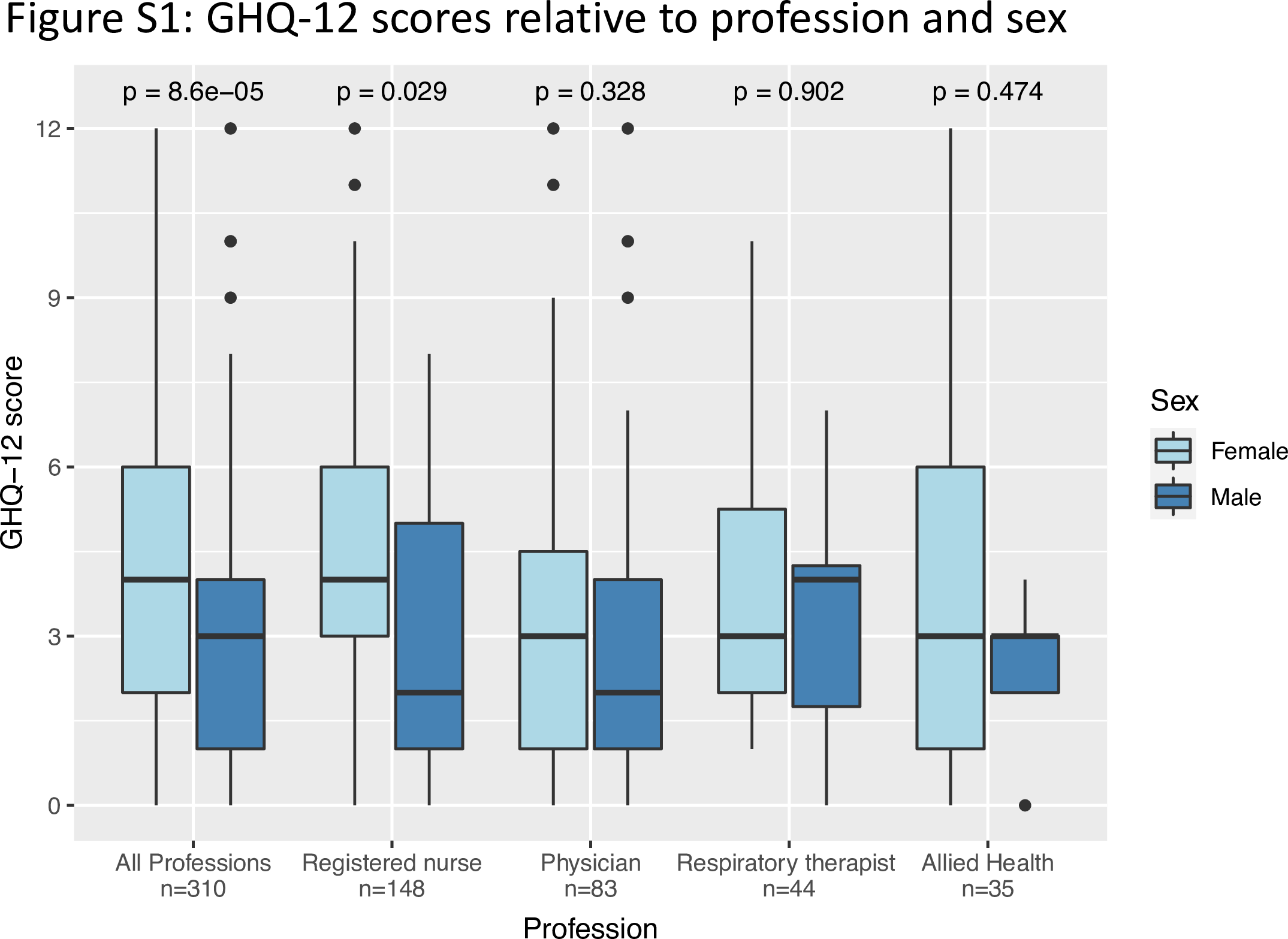

Supplement: S1 Fig — Boxplots of GHQ-12 scores relative to profession and subdivided by sex. P-values compare females and males for each professional group. (TIF) [file pone.0254708.s001.tif]

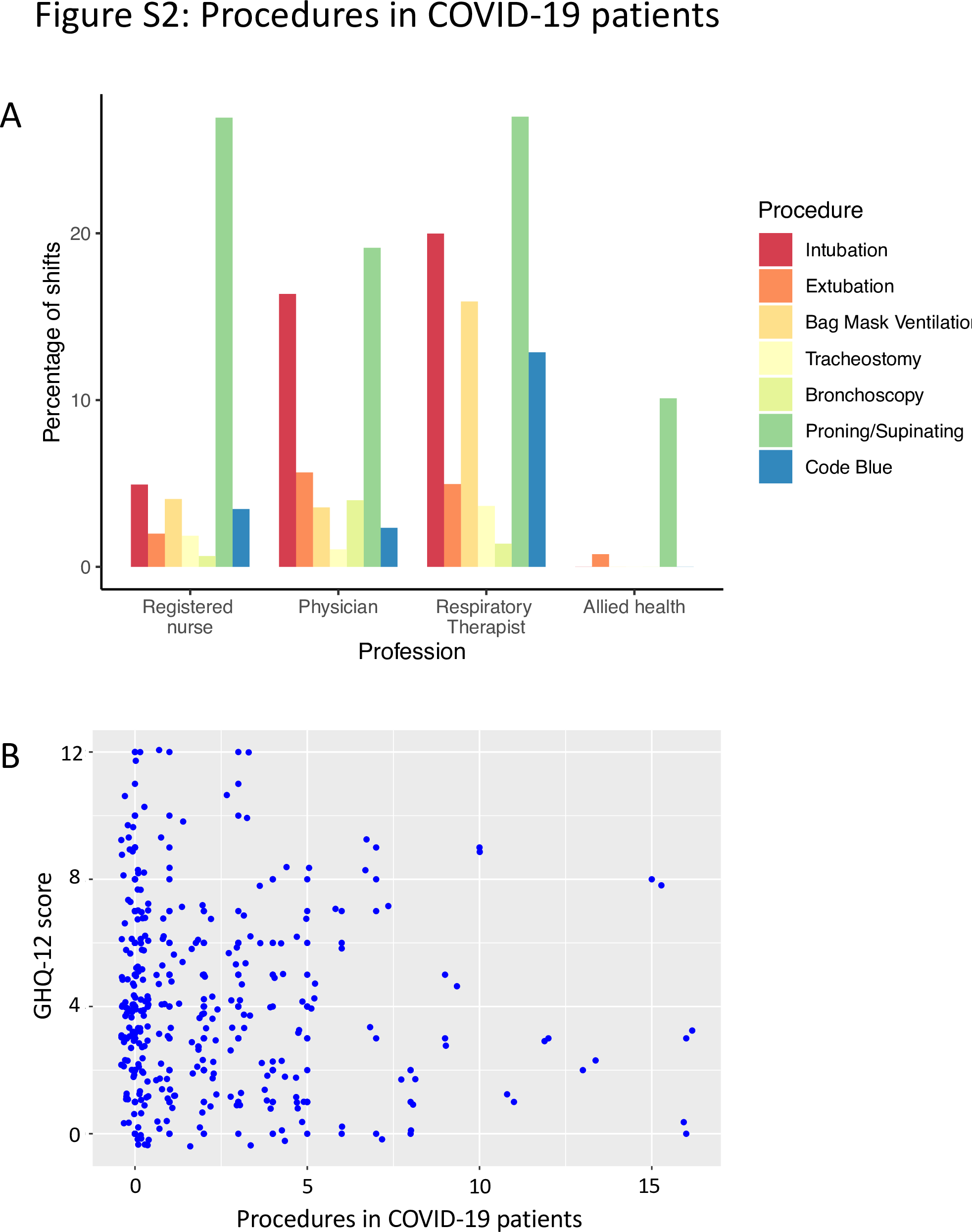

Supplement: S2 Fig — (A) Number of procedures per shift in COVID-19 patients performed by respondents in the previous week. (B) Plot of number of procedures performed in COVID-19 patients in the previous week relative to GHQ-12 score. (TIF) [file pone.0254708.s002.tif]
