# Supplementary material for: Psychosocial distress amongst Canadian intensive care unit healthcare workers during the acceleration phase of the COVID-19 pandemic
Source: PLoS One. 2021 Aug 12;16(8):e0254708. doi: 10.1371/journal.pone.0254708 (PMC8360506; doi:10.1371/journal.pone.0254708)
Supplement: S1 Table — (PDF) [file pone.0254708.s003.pdf]

Table S1: Results of univariate logistic regression for clinically-relevant psychosocial distress (GHQ-12 ≥ 3)

Characteristic	N	OR[1]	95% CI[1]	p-value	q-value[2]
**Age**	310	0.99	0.96, 1.01	0.26	0.42
**Sex**	310			**0.004**	**0.013**
Female		—	—		
Male		0.47	0.28, 0.78		
**Profession**	310			**<0.001**	**0.003**
Allied Health		—	—		
Physician		0.73	0.33, 1.62		
Registered nurse		2.33	1.07, 5.02		
Respiratory therapist		1.19	0.48, 2.95		
**Years of Experience**	309	0.98	0.96, 1.01	0.19	0.35
**Weekly shifts**	302	1.03	0.90, 1.18	0.66	0.77
**Weekly COVID-19 shifts**	304	0.91	0.81, 1.03	0.13	0.35
**ICU COVID-19 patients**	271			0.69	0.77
1) 0		—	—		
2) 1-5		0.73	0.27, 1.81		
3) 6-10		0.62	0.22, 1.66		
4) 11-20		0.72	0.25, 1.94		
5) 21-30		0.25	0.03, 1.77		
**Patients bedspaced outside ICU**	262			0.77	0.77
No		—	—		
Yes		1.10	0.58, 2.14		
**Caring for bedspaced patients outside ICU**	279			0.73	0.77
No		—	—		
Yes		0.89	0.46, 1.77		
**Number of procedures**	278	0.95	0.87, 1.02	0.16	0.35
**Access to PPE**	285	0.90	0.76, 1.05	0.19	0.35
**Access to PPE for AGMP**	277	0.89	0.74, 1.05	0.18	0.35
**Access to sanitizer**	282	0.96	0.78, 1.16	0.68	0.77
**Anxiety re access to PPE/sanitizer**	297	1.25	1.09, 1.43	**<0.001**	**0.004**
**Anxiety re personal risk of infection**	296	1.47	1.27, 1.72	**<0.001**	**<0.001**
**Anxiety re transmission to others**	295	1.30	1.15, 1.49	**<0.001**	**<0.001**
**Knowledge re protecting against infection**	299	0.87	0.67, 1.12	0.28	0.42
**Knowledge re preventing transmission**	299	0.93	0.75, 1.14	0.50	0.69

[1] OR = Odds Ratio, CI = Confidence Interval

[2] False discovery rate correction for multiple testing
