# Supplementary material for: Psychosocial distress amongst Canadian intensive care unit healthcare workers during the acceleration phase of the COVID-19 pandemic
Source: PLoS One. 2021 Aug 12;16(8):e0254708. doi: 10.1371/journal.pone.0254708 (PMC8360506; doi:10.1371/journal.pone.0254708)
Supplement: S1 Appendix — The complete study survey is shown with the exception of the GHQ-12 questionnaire. (PDF) [file pone.0254708.s004.pdf]

Default Question Block

Thank you for agreeing to participate in the COVID-HCW Study. Please complete the demographics questions below.

Age:

[ ]

Sex:

- ○ Male
- ○ Female
- ○ [ ] Other

Self-identified race (optional):

[ ]

Professional Role

- ○ Staff physician
- ○ Trainee physician
- ○ Registered nurse
- ○ Respiratory therapist
- ○ Physiotherapist
- ○ Registered dietician
- ○ Pharmacist

○ [ ] Other

If you are an MD, please list specialty:

[ ]

Years of experience in current professional role since completing training (for MDs, please count from graduating medical school)

[ ]

Province/territory of employment:

- ○ Alberta
- ○ British Columbia
- ○ Manitoba
- ○ New Brunswick
- ○ Newfoundland and Labrador
- ○ Northwest Territories
- ○ Nova Scotia
- ○ Nunavut
- ○ Ontario
- ○ Prince Edward Island
- ○ Quebec
- ○ Saskatchewan
- ○ Yukon

Please consider the following aspects of your life and rate on a scale from (1) to (100) whether they are currently causing you stress:

	0	10	20	30	40	50	60	70	80	90	100
Your work life											
Your home life											
Your finances											
Your physical health											
Your mental health											
Your family's health											

Are there any other significant causes of stress you would like to mention?

Powered by Qualtrics

Version 2 - April 5, 2020

1

In the past week, how many shifts did you work at the hospital? (For MDs working 24-hour shifts, please count as 2 shifts.)

In the past week, how many shifts did you work in which you had direct contact with known or suspected COVID-19 patients?

If you are an RN, how many shifts did you work in which you were the "primary" nurse for known or suspected COVID patients?

2

For the following questions, "COVID-19 patients" refers to known OR suspected COVID-19 cases.

In the past week, approximately how many COVID-19 patients were in the ICUs in your hospital?

- ○ 0
- ○ 1-5
- ○ 6-10
- ○ 11-20

- 21-30
- 31-50
- >50
- I don't know

In the past week, were critically-ill COVID-19 patients bed-spaced to areas outside the regular ICU(s)?

- Yes
- No
- I don't know

3

In the past week, did you take care of ICU-level COVID-19 patients in areas outside the regular ICU(s)?

- Yes
- No

4

In the past week, how many times were you in the room with a COVID-19 patient during the following aerosol-generating medical procedures (AGMP)?

	0	1	2	3	4	5	6	7	More than 7
Intubation	○	○	○	○	○	○	○	○	○
Extubation	○	○	○	○	○	○	○	○	○

Bag mask ventilation	○	○	○	○	○	○	○	○	○
Tracheostomy	○	○	○	○	○	○	○	○	○
Bronchoscopy	○	○	○	○	○	○	○	○	○
Proning	○	○	○	○	○	○	○	○	○
Code blue	○	○	○	○	○	○	○	○	○

In the past week, were you in a room with COVID-19 patients receiving the following (choose all that apply):

☐ Non-invasive ventilation (CPAP/BiPAP)
☐ High flow nasal cannula (Airvo/Optiflow)
☐ Neither

5

In the past week, at any time were you symptomatic with a possible COVID-19 infection?

○ Yes
○ No

Questions about symptoms:

Date of symptom onset:

Month [ ]

Day

What symptoms did you have (choose all that apply)?

- ☐ Fever
- ☐ Headache
- ☐ Dry cough
- ☐ Productive cough
- ☐ Sore throat
- ☐ Runny nose
- ☐ Shortness of breath
- ☐ Myalgias (muscle pains)
- ☐ Loss of taste or smell
- ☐ Loss of appetite
- ☐ Diarrhea
- ☐ ____________ Other

Were you asked to quarantine due to symptoms?

- ○ Yes
- ○ No

Start date of quarantine:

Month

Day

How long were you asked to quarantine for?

- ○ 7 days
- ○ 14 days
- ○ [ ] Other

6

COVID-19 exposure events

In the past week, were you at any time exposed to a known or presumed COVID-19 patient without wearing adequate PPE as stipulated by your hospital (either in the community or at work)?

- ○ Yes
- ○ No

Information about COVID-19 exposure event

Date of exposure:

Month [ ]

Day [ ]

Where did the exposure take place?

- ○ At work
- ○ Outside of work (i.e. at home or in the community)

Describe the exposure:

Was the exposure during an aerosol-generating medical procedure (AGMP)?

- ○ Yes
- ○ No

Which recommended PPE were you NOT wearing during the exposure event?

- ○ Surgical mask
- ○ N95 mask
- ○ Gown
- ○ Gloves
- ○ Protective eye wear
- ○ Face shield
- ○ Boots
- ○ Hair cap
- ○ All PPE
- ○ ______ Other

Were you asked to quarantine after the exposure?

- ○ Yes
- ○ No

Start date of quarantine:

Month

Day

How long were you asked to quarantine for?

- 7 days
- 14 days
- ________ Other

7

COVID-19 Testing

In the past 2 (TWO) weeks have you been test for COVID-19?

- Yes
- No

What was the date of the test?

Month

Day

What was the reason for the test?

- Symptoms
- Exposure to COVID-19 without adequate PPE
- Out of country travel
- Routine screening of healthcare workers
- ________ Other

What was the result of the test?

- Positive
- Negative
- Results pending

Were you asked to quarantine?

- Yes
- No

How long were you asked to quarantine for?

- 7 days
- 14 days
- Other

8

PPE use and availability

Please evaluate the following statements:

In the past week, I always have access to the following in accordance with my hospital policy:

	Strongly disagree	Disagree	Somewhat disagree	Neither agree nor disagree	Somewhat agree	Agree	Strong[illegible] agre[illegible]

Appropriate personal protective equipment (PPE) for routine COVID-19 patient care.	○	○	○	○	○	○	○
Appropriate personal protective equipment (PPE) for aerosol-generating medical procedures (AGMP) in COVID-19 patients.	○	○	○	○	○	○	○
Hand sanitizer for COVID-19 patient care.	○	○	○	○	○	○	○

Please rate your level of anxiety regarding the following issues:

	Not anxious (1)	(2)	(3)	(4)	(5)	(6)	Extremely anxious (7)
The availability of PPE and hand sanitizer.	○	○	○	○	○	○	○
My personal risk of contracting COVID-19.	○	○	○	○	○	○	○

My risk of transmitting COVID-19 to my family and loved ones.	○	○	○	○	○	○	○

9

Knowledge about PPE

Please rate the following:

	Extremely poor (1)	(2)	(3)	(4)	(5)	(6)	Extremely good (7)
My level of knowledge about protecting myself from COVID-19.	○	○	○	○	○	○	○
My level of knowledge about preventing transmission of COVID-19 to my family and loved ones.	○	○	○	○	○	○	○

10

Did you take any of the following medications last week?

- ☐ Hydroxychloroquine
- ☐ Chloroquine
- ☐ Kaletra (lopinavir/ritonavir)
- ☐ Resmdesivir
- ☐ Azithromycin

- ☐ Inhaled steroids
- ☐ Oral steroids
- ☐ None of the above

Was the medication taken for:

- ☐ Coronavirus treatment
- ☐ Coronavirus prevention
- ☐ Other medical issue

Block 10

Overall Work Experience

How could your experience at work in the past week be improved?

Powered by Qualtrics
