# Supplementary material for: Psychosocial distress amongst Canadian intensive care unit healthcare workers during the acceleration phase of the COVID-19 pandemic
Source: PLoS One. 2021 Aug 12;16(8):e0254708. doi: 10.1371/journal.pone.0254708 (PMC8360506; doi:10.1371/journal.pone.0254708)
Supplement: S2 Appendix — (PDF) [file pone.0254708.s005.pdf]

COVID-HCW: Supplementary methods

Survey Design

The survey was designed as the entry questionnaire for a longitudinal study of exposure and transmission risks for COVID-19 infection in ICU HCW. It was built in an expedited fashion to capture data prior to the peak of the first wave of the pandemic in Canada. Four domains were identified as potential contributors to HCW psychosocial distress in emerging infectious disease outbreaks based on literature review: (1) demographics (2) exposure to infected patients, (3) access to PPE and (4) anxieties regarding availability of PPE, risk of infection, and risk of transmitting infection to others. To assess psychosocial distress levels, participants completed the General Health Questionnaire 12-item scale (GHQ-12), a screening tool that focuses on breaks in normal functioning rather than life-long traits (9). It has been validated in workplace studies in English and French (10–12) as well as in studies of HCW during emerging disease outbreaks (13,14). Finally, participants were asked to rate the level of stress associated with 6 “life domains”: work life, home life, finances, physical health, mental health, and family health on a scale of 0-100.

Item generation and reduction were performed using the modified Delphi method. Five clinicians (physicians and nurses) reviewed the questionnaire for ease of administration, flow and salience, with iterative adaption based on their feedback. Pre-testing was not performed due to time constraints. The survey was translated into French by the research team and participants could choose to participate in either language.

Items were not randomized or alternated. Certain items were conditionally displayed based on previous answers. Responses to all questions were voluntary and completeness checks were not enforced. There were no incentives offered. Respondents could review responses prior to submission. Only submitted surveys were analysed. View rates and recruitment rates were not quantified. The survey is available in Appendix I.

Survey Administration

The survey was administered online through a secure Qualtrics platform (www.qualtrics.com). The survey target population was Canadian ICU HCW including physicians, registered nurses, respiratory therapists, and allied healthcare (physiotherapists, occupational therapists, dieticians, pharmacists, social workers, and nurses’ aids). In order to capture a broad cross-section of ICU HCW at multiple hospitals the survey was open, however dissemination of the online study advertisement was limited to the Canadian Critical Care Trials Group, the Canadian Community ICU Research Network, the Canadian Critical Care Society, and the Critical Care Nurses Association along with related social media groups. Distribution began on April 6th, 2020

and all responses were received by April 30th, 2020, prior to the peak of the first wave of the pandemic in Canada (15). Participation was voluntary and without incentive. Consent was provided via e-consent. Due to the self-selected and non-probabilistic nature of the sample, invitations and response rates could not be quantified. Unique participants were identified via email address, which was stored securely and separately from response data. Cookies and IP checks were not used.

Data Analysis

Data are described as mean and standard deviation (or median and interquartile range (IQR)) for continuous variables and number and percentage for categorical variables. Continuous variables were compared using the student t-test or Kruskal-Wallis and Wilcoxon rank sum tests. Categorical variables were compared using Chi square probability distribution (χ^2). Correlations were performed using Pearson's correlation coefficient (r). Data from 7-point Likert scales were treated as continuous variables.

Response categories on the GHQ-12 questionnaire were coded using the 0-0-1-1 scoring method recommended by Goldberg (9). A threshold of ≥ 3 points on the GHQ-12 has been identified as an appropriate cutoff for mental health diagnosis screening (10,14) and a marker of clinically-relevant psychosocial distress (13,16).

Independent predictors of clinically-relevant psychosocial distress (GHQ-12 ≥ 3) were assessed using univariate logistic regression models. Explanatory variables, including demographic variables, COVID-19 exposure variables, access to PPE and sanitizer variables, anxiety variables, and knowledge variables were selected for relevance to the outcome. For multivariate analysis, forward and backward stepwise regression was performed to achieve the lowest Akaike Information Criteria (AIC) with each variable in the final model showing a p value <0.05. All analysis was performed using R software v 3.6.2 (17).
